# Hypervirulent carbapenem-resistant *Klebsiella pneumoniae* causing highly fatal meningitis in southeastern China

**DOI:** 10.3389/fpubh.2022.991306

**Published:** 2022-10-17

**Authors:** Na Huang, Huaiyu Jia, Beibei Zhou, Cui Zhou, Jianming Cao, Wenli Liao, Shixing Liu, Lingbo Wang, Liqiong Chen, Lijiang Chen, Tieli Zhou, Jianzhong Ye

**Affiliations:** ^1^Department of Clinical Laboratory, Key Laboratory of Clinical Laboratory Diagnosis and Translational Research of Zhejiang Province, The First Affiliated Hospital of Wenzhou Medical University, Wenzhou, China; ^2^School of Laboratory Medicine and Life Science, Wenzhou Medical University, Wenzhou, China

**Keywords:** *Klebsiella pneumoniae*, meningitis, hypervirulence, carbapenem-resistant, infection

## Abstract

*Klebsiella pneumoniae* (*K. pneumoniae*) is one of the most common causes of bacterial meningitis worldwide. The purpose of this study was to investigate the clinical and microbiological characteristics of *K. pneumoniae* meningitis, as well as the association of antimicrobial resistance, virulence, and patient prognosis. The clinical data of patients with *K. pneumoniae* meningitis from 2014 to 2020 in a tertiary teaching hospital were retrospectively evaluated. Antimicrobial susceptibility profiles were performed by the agar dilution method and broth microdilution method. The isolates were detected for virulence-related genes, resistance genes, capsular serotypes, and molecular subtypes. A total of 36 individuals with *K. pneumoniae* meningitis were included in the study, accounting for 11.3% (36/318) of all cases of bacterial meningitis. Of the 36 available isolates, K1, K47, and K64 were tied for the most frequent serotype (7/36, 19.4%). MLST analysis classified the isolates into 14 distinct STs, with ST11 being the most common (14/36, 38.9%). Carbapenem resistance was found in 44.4% (16/36) of the isolates, while hypervirulent *K. pneumoniae* (HvKP) was found in 66.7% (24/36) of the isolates. The isolates of hypervirulent carbapenem-resistant *K. pneumoniae* (Hv-CRKP) were then confirmed to be 36.1% (13/36). Importantly, individuals with meningitis caused by Hv-CRKP had a statistically significant higher mortality than the other patients (92.3%, 12/13 vs. 56.5%, 13/23; *P* < 0.05). The high percentage and fatality of *K. pneumoniae*-caused meningitis, particularly in Hv-CRKP strains, should be of significant concern. More effective surveillance and treatment solutions will be required in future to avoid the spread of these life-threatening infections over the world.

## Introduction

Bacterial meningitis is a potentially fatal cerebral illness that necessitates lengthy hospital stays and high expenses due to inadequate healthcare delivery (1, 2). The significant mortality and morbidity of bacterial meningitis have prompted widespread and severe concerns all over the world in recent years (2). Bacterial meningitis causes a variety of clinical symptoms, including fever, headache, nausea, vomiting, neck stiffness, or altered mental status, which can lead to herniation, coma, and even death (3, 4).

*Klebsiella pneumoniae* (*K. pneumoniae*) is an emerging cause of bacterial meningitis worldwide (5). Previously, *K. pneumoniae* was an increasingly frequent pathogen in Taiwan, and it was even the first pathogen engaged in adult community-acquired bacterial meningitis (CABM), with a prevalence rate of up to 68.3% among gram-negative pathogenic organisms (6). Fortunately, all third- and fourth-generation cephalosporins were effective against the *K. pneumoniae* strains, and none of the clinical isolates were shown to be an extended-spectrum β-lactamase-producing pathogen (6). The fast development of antimicrobial resistance bacteria, on the contrary, poses a severe public health risk. One recent research found that *K. pneumoniae* strains isolated from cerebrospinal fluid (CSF) were resistant to the majority of first-line antibiotics including carbapenems, with the exception of tigecycline and colistin (7). Carbapenem-resistant *K. pneumoniae* (CRKP) is classified as a “critical” category on the WHO global priority pathogen list (8). KPC-carrying strains are most common in China and are frequently associated with other types of β-lactamases (e.g., ESBLs) (7, 9). *Klebsiella pneumoniae* has been separated into two essentially nonoverlapping populations over the past three decades: classic *K. pneumoniae* (cKP) and hypervirulent *K. pneumoniae* (HvKP), both of which may produce severe invasive and disseminated infections (10, 11). HvKP strains are distinguished from cKP pathogens by genes on a large virulence plasmid or on chromosomal islands. One trustworthy study used the virulence genes *peg-344* and *iucA* to distinguish between HvKP and cKP strains with a sensitivity of 0.94 and specificity of 1.0 (12). Furthermore, *K. pneumoniae* strains causing meningitis contain distinct resistance genes, capsular serotypes, virulence genes, and related clones, which might eventually lead to plasmid-mediated resistance and virulence transmission, which can have a negative influence on patient prognosis (13). However, few research has been conducted to investigate the characteristics of *K. pneumoniae* meningitis in depth.

In this study, we conducted a retrospective survey of *K. pneumoniae* meningitis in a tertiary teaching hospital of southeastern China during a 7-year period, systematically investigating the clinical, microbiological, and molecular epidemiological characteristics of *K. pneumoniae* meningitis, and comprehensively evaluating the risk factors that affect patients' survival, to provide some data for optimizing treatment of *K. pneumoniae* meningitis.

## Materials and methods

### Study patients

A total of 318 patients with *K. pneumoniae* meningitis were included between 2014 and 2020 in the First Affiliated Hospital of Wenzhou Medical University, which is a large tertiary teaching hospital located in Southeast China with 4,100 beds and more than 160,000 inpatients per year. Inclusion criteria for *K. pneumoniae* meningitis were as follows: (1) positive *K. pneumoniae* CSF culture; (2) suggestive symptoms of meningitis including fever, headache, vomiting, unconscious, muscle tone changes, and abnormal clinical laboratory examination findings including white blood cell count, procalcitonin, C-reactive protein, percentage of neutrophils in peripheral blood, red blood cell count, white blood cell count, sugar, chlorine, and protein content in CSF; (3) the case data and follow-up data of patients were complete.

### *Klebsiella pneumoniae* isolation and identification

CSF samples were isolated from meningitis patients and cultured using a BacT/ALERT 3D microbial detection system (bioMerieux, Durham, NC) before being identified as *K. pneumoniae* in a matrix-assisted laser desorption/ionization time-of-flight mass spectrometry system (MALDI-TOF/MS; bioMérieux, Lyons, France). The isolates were kept at −80°C for further research. Duplicate strains isolated from the same patient were removed from the strain collecting process.

### Antimicrobial susceptibility testing

The minimum inhibitory concentrations (MICs) of ampicillin, aztreonam, ceftriaxone, ceftazidime, cefepime, imipenem, ciprofloxacin, levofloxacin, gentamicin, tobramycin, sulfamethoxazole/trimethoprim, nitrofurantoin, colistin, and tigecycline were detected by the agar dilution method and broth microdilution method. The data were interpreted by the latest guidelines published by the Clinical and Laboratory Standards Institute (CLSI 2021, Pittsburgh, PA, USA). The MIC of colistin was determined with the broth microdilution method and interpreted using the recommendation of the European Committee on Antimicrobial Susceptibility Testing clinical breakpoints (EUCAST, http://www.eucast.org/). *Escherichia coli* ATCC 25922 was used as the control strain for antimicrobial susceptibility testing.

### Detection of capsular serotypes gene, virulence genes, and resistance genes

Genomic DNA of *K. pneumoniae* strains was extracted using the Biospin Bacterial Genomic DNA Extraction kit (BioFlux, Tokyo, Japan). Subsequently, specific primers (Supplementary Table 1) were used to amplify the capsular serotype gene (*wzi*), virulence genes (*peg-344, iroB, iucA, rmpA*, and *rmpA2*), carbapenem resistance genes (*bla*_KPC − 2_, *bla*_NDM_, *bla*_IMP_, *bla*_VIM_, and *bla*_OXA − 48_), and ESBLs resistance genes (*bla*_SHV_, *bla*_TEM_, *bla*_CTX − M−1_, *bla*_CTX − M−2_, *bla*_CTX − M−9_, *bla*_CTX − M−25_, and *bla*_CTX − M−65_), and membrane porins encode resistance genes (OmpK35, OmpK36, and OmpK37).

### Differentiation of HvKP and cKP strains

According to a credible study, we employed two virulence genes, *peg-344* and *iucA*, to separate HvKP and cKP strains with a sensitivity of 0.94 and specificity of 1.0, demonstrating the efficacy of gene markers for accurately identifying HvKP strains (12).

### Multilocus sequence typing

MLST analyses of the *K. pneumoniae* isolates were performed by amplifying seven housekeeping genes (*gapA, mdh, phoE, tonB, infB, pgi*, and *rpoB*). Sequence types were allocated by searching the database (https://blast.ncbi.nlm.nih.gov/Blast.cgi?CMD=Web&PAGE_TYPE=BlastHome) using the given methods.

### Statistical analysis

The statistical analysis was calculated using SPSS 22.0 software (IBM, Armonk, NY, USA). Continuous data were reported as mean ± standard deviation (mean ± SD) or median [inter-quartile range (IQR)] and analyzed using unpaired Student's *t*-test (two-tailed), while categorical variables were given as percentages and compared using the chi-square test (two-tailed). Multivariate logistic regression analysis was used to explore the independent risk factors for death from *K. pneumoniae* meningitis. A *P*-value of <0.05 was considered as statistically significant.

### Ethics approval

This study conformed to the Declaration of Helsinki and was approved by the Ethics Committee of the First Affiliated Hospital of Wenzhou Medical University (Issuing No. 2021R094). Written informed consent for inclusion from each patient was waived by the Ethics Committee of the First Affiliated Hospital of Wenzhou Medical University because this was a retrospective study, and no study-related interventions were included.

## Results

### *Klebsiella pneumoniae* strains isolated from cerebrospinal fluid

In this study, a total of 318 cases of bacterial meningitis were detected at the First Affiliated Hospital of Wenzhou Medical University (Wenzhou, China) from 2014 to 2020, and 36 *K. pneumoniae* meningitis were eventually screened according to the inclusion criteria (Supplementary Figure 1). The percentage of *K. pneumoniae* isolates ranked fourth (11.3%, 36/318) among all pathogens and second to *Acinetobacter baumannii* (14.51%, 46/318) among gram-negative pathogens.

### Clinical characteristics of the 36 *K. pneumoniae* meningitis cases

There were 29 male patients and seven female patients among the 36 *K. pneumoniae* meningitis patients, with an average age of 54 years old (IQR 44, 62), an ICU admission rate of 58.3% (21/36), a mechanical ventilation rate of 75.0% (27/36), and a fatality rate of 69.4% (25/36). About 27.8% (10/36) of the meningitis cases were community-acquired infection, while 72.2% (26/36) were acquired in the hospital. The detailed clinical characteristics of the 36 cases are summarized in Table 1 and Supplementary Table 2. When compared to the survival group, the death group demonstrated statistically significant differences in vomiting (*P* = 0.015), unconsciousness (*P* = 0.010), length of stay days (*P* = 0.016), bacteremia (*P* = 0.025), ICU admission (*P* = 0.025), mechanical ventilation (*P* = 0.012), Glasgow coma scale (GCS) score (*P* = 0.001), C-reactive protein (*P* = 0.008), and protein in CSF profiles (*P* = 0.014; Table 1). When compared to the Hv-CRKP group, the non-Hv-CRKP group showed differences in age (*P* = 0.034) and diabetes mellitus (*P* = 0.025, Supplementary Table 3).

**Table 1:** Clinical characteristics of the 36 *K. pneumoniae* meningitis cases.

**Clinical characteristics**	**Total (*n* = 36)**	**Available patients (*****n*** = **36)**		***P_1_***	***P_2_***
		**Death group (*n* = 25)**	**Survival group (*n* = 11)**		
**Demographic data**
Age (years), median (IQR)	54, (44, 62)	52, (39, 61)	55, (49, 65)	0.346	
Gender, *n* (%)				0.167	
Male	29 (80.6)	22 (88.0)	7 (63.6)		
Female	7 (19.4)	3 (12.0)	4 (36.4)		
**Underlying conditions**, ***n*** **(%)**
Hypertension	13 (36.1)	9 (36.0)	4 (36.4)	1.000	
Diabetes mellitus	12 (33.3)	6 (24.0)	6 (54.5)	0.124	
History of surgery	11 (30.6)	6 (24.0)	5 (45.5)	0.252	
Head trauma	11 (30.6)	9 (36.0)	2 (18.2)	0.439	
**Clinical symptoms**, ***n*** **(%)**
Fever	11 (30.6)	8 (32.0)	3 (27.3)	1.000	
Headache	8 (22.2)	8 (32.0)	0	0.076	
Vomiting	11 (30.6)	11 (44.0)	0	**0.015**	0.998
Unconscious	19 (52.8)	17 (68.0)	2 (18.2)	**0.010**	0.999
Muscle tone changes	12 (33.3)	11 (44.0)	1 (9.1)	0.059	
Length of stay (Days), median (IQR)	20 (8, 38)	36 (20, 55)	36 (21, 46)	**0.016**	1.000
**Extrameningeal infections**, ***n*** **(%)**
Bacteremia	14 (38.9)	13 (52.00)	1 (9.1)	**0.025**	0.999
Pneumonia	19 (52.8)	14 (56.0)	5 (45.5)	0.721	
Liver abscess	2 (5.6)	1 (4.00)	1 (9.1)	0.524	
Brain abscess	6 (16.7)	4 (16.00)	2 (18.2)	1.000	
Stay in ICU	21 (58.3)	18 (72.0)	3 (27.3)	**0.025**	0.999
Mechanical ventilation	27 (75.0)	22 (88.0)	5 (45.5)	**0.012**	0.999
GCS, median (IQR)	12.5 (1, 14.50)	15 (14, 15)	8.5 (5, 11.75)	**0.001**	0.999
**Inflammation index, median (IQR)**
White blood cell count (WBC,10^9^/L)	12.44 (9.72, 14.82)	13.08 (10.00, 15.69)	11.49 (8.59, 13.47)	0.503	
Procalcitonin (PCT, ug/L)	1.15 (0.14, 12.18)	1.88 (0.18, 26.96)	0.23 (0.09, 9.43)	0.238	
C reactive protein (CRP, mg/L)	90.00 (72.40, 90)	89.10 (49.96, 90)	20.90 (12.15, 83.55)	**0.008**	1.000
Percentage of neutrophils	0.87 (0.83, 0.91)	0.87 (0.83, 0.91)	0.87 (0.72, 0.89)	0.283	
**CSF profiles, median (IQR)**
RBC count (per μL)	590 (111, 2017)	980 (111, 2230)	260 (37, 790)	0.138	
WBC count (per μL)	3500 (126, 19600)	4680 (107, 27600)	1320 (330, 3520)	0.350	
Sugar (mmol/L)	1.11 (1.11, 3.40)	1.11 (1.11, 3.8)	1.41 (1.11, 3)	0.808	
Chlorine (mmol/L)	116 (110, 121)	116 (110, 125)	116 (109, 119)	0.860	
Protein (g/L)	6000 (2934, 13961)	7060 (5004, 16566)	2934 (1730, 5335)	**0.014**	1.000

ICU, Intensive Care Unit; GCS, Glasgow Coma Scale; RBC, Red Blood Cell; WBC, White Blood Cell; P_1_, P-value at univariate analysis; P_2_, P-value in multivariate logistic regression analysis. The bold values indicate statistically significance.

### Antimicrobial susceptibility profiles

The antimicrobial resistance information and detailed MICs of the 36 isolates are listed in Table 2 and Supplementary Table 4 in the supplemental material. Generally, the isolates showed high resistance rates to ampicillin (AMP), ceftriaxone (CRO), ceftazidime (CAZ), cefepime (FEP), nitrofurantoin (NIT), ciprofloxacin (CIP), levofloxacin (LEV), gentamicin (GEN), and fosfomycin (FOS), and 47.2% (17/36) of the isolates were identified as multi-drug resistant (MDR), which were defined as non-susceptible to three or more different antimicrobial categories. Notably, 41.7% (15/36) of the isolates were resistant to imipenem and meropenem, whereas 44.4% (16/36) were resistant to ertapenem. Colistin and tigecycline were effective against all 36 isolates. There were no significant differences in antimicrobial resistance rates between the surviving and mortality groups (Table 2).

**Table 2:** Antimicrobial resistance patterns of 36 *K. pneumoniae* strains.

**Antimicrobial resistance, *n* (%)**	**Total (*n* = 36)**	**Available patients or isolates (*****n*** = **36)**		***P*-values**
		**Death group (*n* = 25)**	**Survival group (*n* = 11)**	
AMP, ampicillin	36 (100)	25 (100)	11 (100)	–
CRO, ceftriaxone	17 (47.2)	14 (56.0)	3 (27.3)	0.156
CAZ, ceftazidime	15 (41.7)	12 (48.0)	3 (27.3)	0.295
FEP, cefepime	15 (41.7)	12 (48.0)	3 (27.3)	0.295
NIT, nitrofurantoin	22 (61.1)	17 (68.0)	5 (45.5)	0.273
CIP, ciprofloxacin	17 (47.2)	13 (52.0)	4 (36.4)	0.481
LEV, levofloxacin	15 (41.7)	12 (48.0)	3 (27.3)	0.295
GEN, gentamicin	15 (41.7)	12 (48.0)	3 (27.3)	0.295
TOB, tobramycin	12 (33.3)	10 (40.0)	2 (18.2)	0.268
SXT, sulfamethoxazole	8 (22.2)	7 (28.0)	1 (9.1)	0.388
FOS, fosfomycin	24 (66.7)	18 (72.0)	6 (54.5)	0.446
ETP, ertapenem	16 (44.4)	13(52.0)	3 (27.3)	0.277
IPM, imipenem	15 (41.7)	12 (48.0)	3 (27.3)	0.295
MEM, meropenem	15 (41.7)	12 (48.0)	3 (27.3)	0.295
COL, colistin	0	0	0	–
TGC, tigecycline	0	0	0	–
MDR strains	17 (47.2)	14 (56.0)	3 (27.3)	0.156

Date are expressed as numbers (%) unless otherwise stated; MDR, multidrug resistance.

### Molecular epidemiological characteristics of the 36 *K. pneumoniae* strains

**Figure 1:**
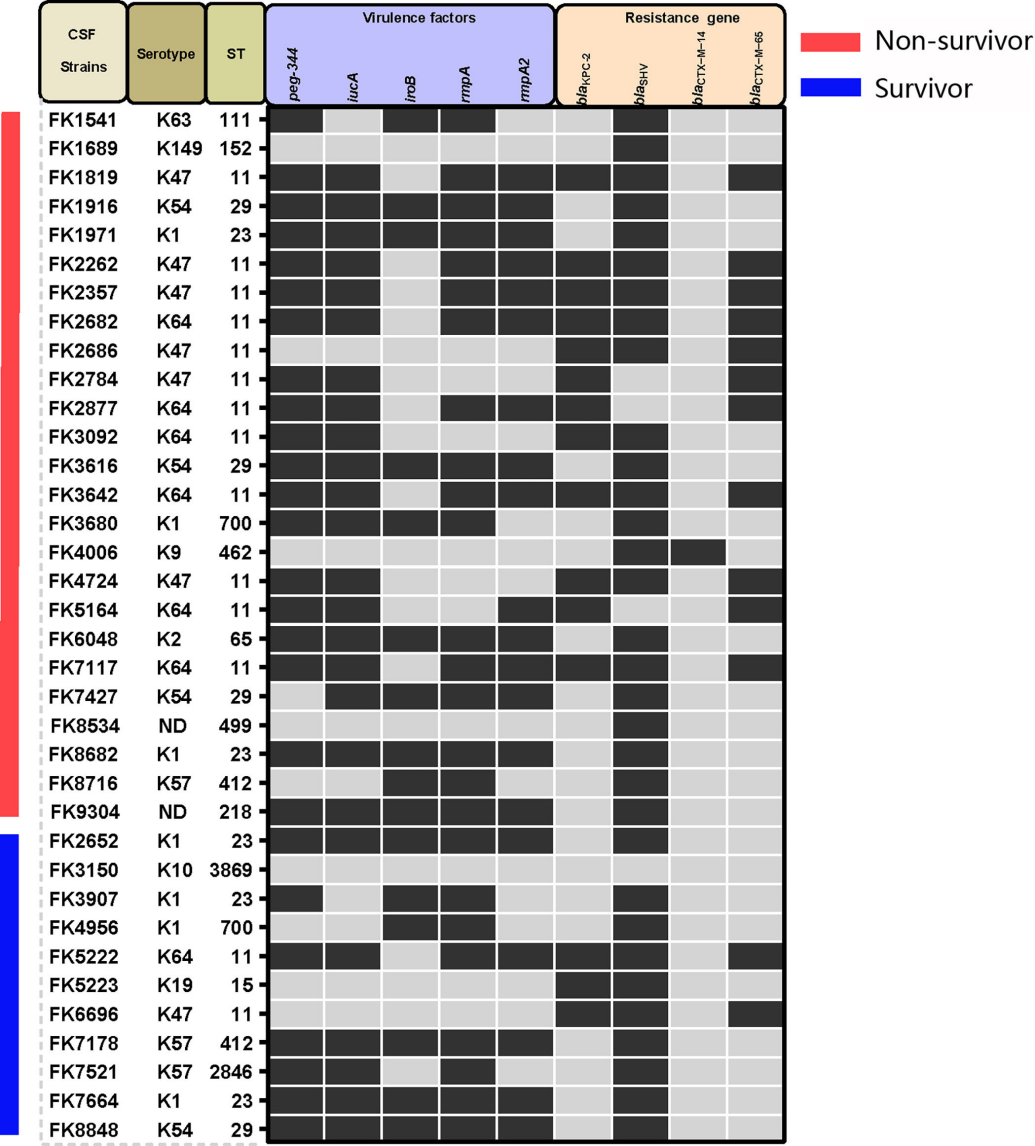
Capsular serotypes, virulence genes, and resistance genes found in the *K. pneumoniae* strains in this study. Positive is represented by black squares, whereas negative is represented by gray squares.

High predominance of capsular serotypes was detected in K1 (7/36, 19.4%), K47 (7/36, 19.4%), K64 (7/36, 19.4%), K54 (4/36, 11.1%), and K57 (3/36, 8.3%), whereas K2, K9, K10, K19, K63, and K149 showed relatively low ratios of <10%. There were no statistically significant differences in capsular serotype distribution between strains isolated from patients with poor prognosis and those with good prognosis (Table 3 and Figure 1). MLST classified the 36 *K. pneumoniae* isolates into 14 distinct sequence types (STs), with ST11 being the most common (14/36, 38.9%), followed by ST23 (5/36, 13.9%), ST29 (4/36, 11.1%), and other STs (Figure 1 and Supplementary Figure 2).

**Table 3:** Molecular epidemiology of 36 *K. pneumoniae* strains.

**Molecular epidemiology**	**Total (*n* = 36)**	**Available patients or isolates (*****n*** = **36)**		***P*-values**
		**Death group (*n* = 25)**	**Survival group (*n* = 11)**	
**Capsular serotypes**, ***n*** **(%)**
K1	7 (19.4)	3 (12.0)	4 (36.4)	0.167
K2	1 (2.8)	1 (4.0)	0	1.000
K9	1 (2.8)	1 (4.0)	0	1.000
K10	1 (2.8)	0	1 (9.1)	0.306
K19	1 (2.8)	0	1 (9.1)	0.306
K47	7 (19.4)	6 (24.0)	1 (9.1)	0.400
K54	4 (11.1)	3 (12.0)	1 (9.1)	1.000
K57	3 (8.3)	1 (4.0)	2 (18.2)	0.216
K63	1 (2.8)	1 (4.0)	0	1.000
K64	7 (19.4)	6 (24.0)	1 (9.1)	0.400
K149	1 (2.8)	1 (4.0)	0	1.000
Non-typeable	2 (5.6)	2 (8.0)	0	1.000
**Virulence genes**, ***n*** **(%)**
*peg-344*	25 (69.4)	18 (72.0)	7 (63.6)	0.703
*iucA*	24 (66.7)	18 (72.0)	6 (54.5)	0.446
*iroB*	16 (44.4)	10 (40.0)	6 (54.5)	0.483
*rmpA*	25 (69.4)	17 (68.0)	8 (72.7)	1.000
*rmpA2*	20 (55.6)	15 (60.0)	5 (45.5)	0.483
**Antimicrobial resistance gene**, ***n*** **(%)**
*bla*_KPC − 2_	15 (41.7)	12 (48.0)	3 (27.3)	0.295
*bla*_CTX−*M*−14_	1(2.8)	1 (4.0)	0	1.000
*bla*_CTX−*M*−65_	13 (36.1)	11 (44.0)	2 (18.2)	0.259
*bla*_SHV_	32 (88.9)	22 (88.0)	10 (90.9)	1.000

### Distribution of virulence and antimicrobial resistance determinants

Virulence-related genes, including *peg-344, iucA, iroB, rmpA*, and *rmpA2*, were found in proportions of 69.4 (25/36), 66.7 (24/36), 44.4 (16/36), 69.4 (25/36), and 55.6% (20/36), respectively, and no significant differences in virulence distribution were found between strains of the two different prognostic groups (Table 3 and Figure 1) Furthermore, the *bla*_KPC − 2_ gene was found in 15 out of the 36 isolates, whereas *bla*_CTX−*M*−14_, *bla*_CTX−*M*−65_, and *bla*_SHV_ were found in one isolate, 13 isolates, and 32 isolates, respectively. The OmpK36 and OmpK37 mutation caused FK3616 to be resistant to carbapenems drugs. Similarly, there was no statistically significant difference in the distribution of antimicrobial resistance genes between the death and survival groups (Table 3 and Figure 1).

### Hypervirulent carbapenem-resistant *K. pneumoniae* was associated with high patient mortality

**Figure 2:**
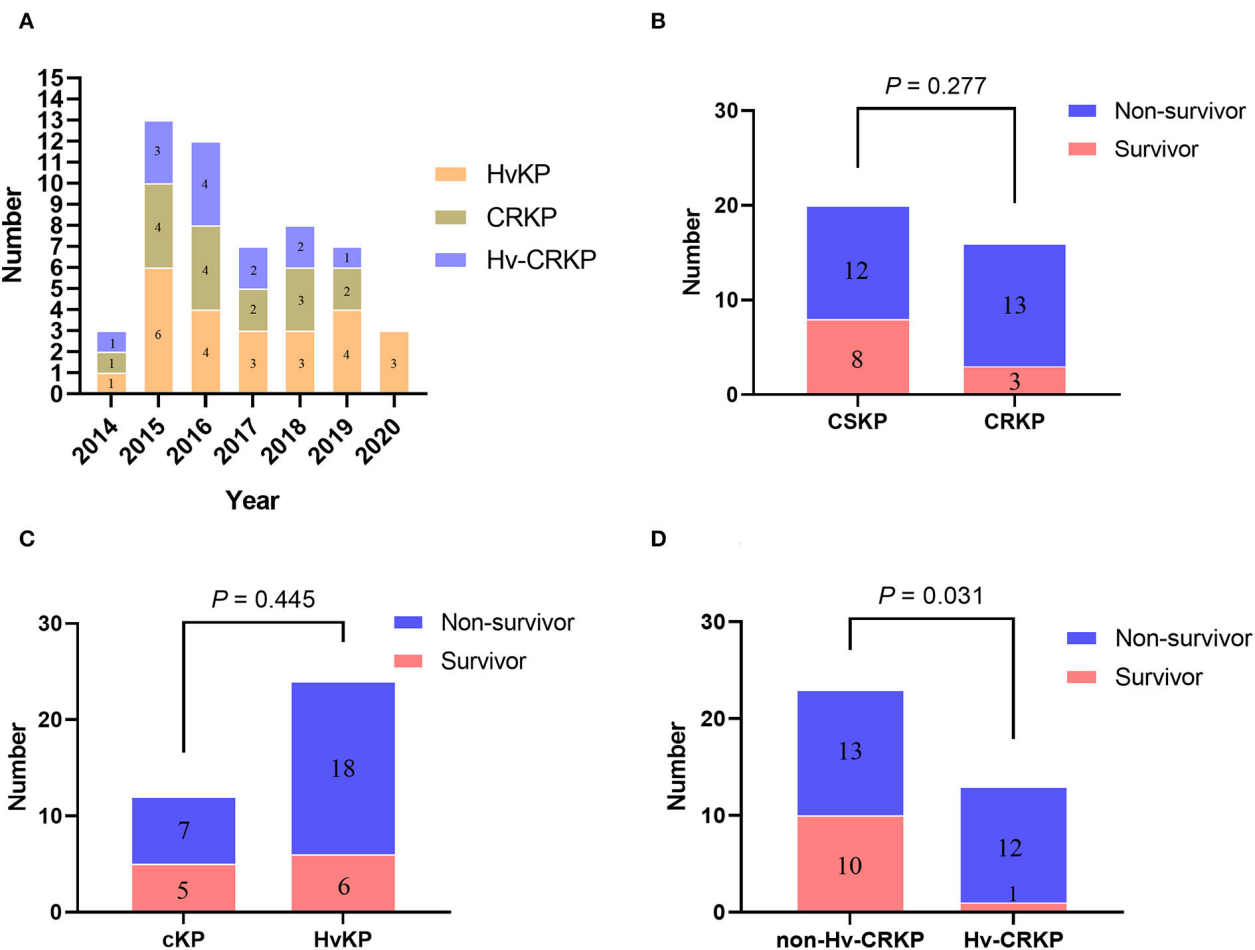
Time distribution and survival rate characteristics of the strains. **(A)** The number of specific strains detected in each year; **(B)** Comparison of mortality between patients infected with CSKP and CRKP; **(C)** Comparison of mortality between patients infected with cKP and HvKP; **(D)** Comparison of mortality between patients infected with non-Hv-CRKP and Hv-CRKP.

We identified the *peg-344* and *iucA* carrying strains as hypervirulence according to one reliable preliminary research with highest efficiency of a sensitivity of 0.94 and specificity of 1.0 (12). Twenty-four isolates were eventually classified as hypervirulent *K. pneumoniae* (HvKP), and 12 isolates were classified as classic *K. pneumoniae* (cKP, Supplementary Table 4). However, only nine strains were identified as hypermucoviscosity phenotype using the traditional string test, and the consistency between hypervirulence and hypermucoviscosity phenotype was only 20.8% (5/24). Furthermore, 16 isolates were identified as carbapenem-resistant *K. pneumoniae* (CRKP) based on antimicrobial susceptibility profiles and 20 isolates as carbapenem-susceptible *K. pneumoniae* (CSKP, Table 2 and Supplementary Table 4). Thus, by overlapping the 24 HvKP strains and 16 CRKP strains, 13 hypervirulent carbapenem-resistant *K. pneumoniae* (Hv-CRKP) isolates were identified, while the other 23 isolates were designated as non-Hv-CRKP strains. Time distributions of the HvKP, CRKP, and Hv-CRKP isolates from 2014 to 2020 are shown in Figure 2A. There were no statistically significant differences in patient mortality between the CRKP and CSKP groups (Figure 2B), nor between HvKP and cKP groups (Figure 2C). However, the Hv-CRKP group had a much greater death rate (92.3%, 12/13) than the non-Hv-CRKP group (56.5%, 13/23, Figure 2D).

## Discussion

Bacterial meningitis, which has a high morbidity and fatality rate, has emerged as a critical public health hazard in recent years (5, 14). This study comprehensively explored the clinical, microbiological, and molecular epidemiological characteristics of *K. pneumoniae*-induced meningitis in a tertiary teaching hospital in China from 2014 to 2020.

In our current study, we discovered a relatively high prevalence of *K. pneumoniae* (11.3%, 36/318) in bacterial meningitis, which is consistent with the epidemiological data from some specific geographic areas (i.e., the Netherlands and Taiwan), where *K. pneumoniae* is commonly described as the primary and predominant meningitis-related pathogens (15, 16). Unfortunately, the fatality rate for the 36 *K. pneumoniae* meningitis patients reached up to 69.4% (25/36). Male gender, advanced age, hypertension, diabetes mellitus, history of surgery, head trauma, and intracerebral hemorrhage were all common among the bacterial meningitis patients, as reported by Lien et al. (17). Only the death group had clinical signs of vomiting and headache, which might be attributed to central nervous system impairment. When compared to the survival group, the death group had a significantly higher frequency of mechanical ventilation and peripheral deep vein catheter operation, an ICU admission rate, and lower GCS score. The death group also had a higher C-reactive protein (CRP) level of blood and protein level of CSF profiles, which was consistent with previous research (18, 19). One study found that Hv-CRKP can be killed by neutrophil extracellular traps (NETs) in diabetics because neutrophils isolated from patients with type 2 diabetes mellitus exhibited enhanced NET formation (20), which may explain why the Hv-CRKP strain is rare in diabetics. In this study, we found that bacteremia is closely related to meningitis patients with poor prognosis, 92.9% (13/14) of the patients with bacteremia and meningitis died, and bacteremia followed by meningitis was identified in 64.3% (9/14) of patients, indicating that bacteremia is an important cause of subsequent meningitis. Our other unpublished findings indicated that outer membrane protein A (OmpA) may help *K. pneumoniae* cross the blood–brain barrier and cause meningitis in bacteremia patients. Because interactions between hosts and pathogens lead to a different clinical outcomes during bacterial infections, clinicians should pay special attention to patients who exhibit the features listed above.

There are at least 78 capsular serotypes of *K. pneumoniae* strains, with capsule types K1 and K2 being particularly pathogenic (21). The prevalence of K1 and K2 in our study was 19.4 (7/36) and 2.8% (1/36), respectively, which was essentially consistent with the previous report (7). ST11 was determined to be the most common type in the MLST study, with a rate of 38.9% (14/36), which was similar to a previous report (22). ST11 is the most dominant *bla*_KPC − 2_-bearing *K. pneumoniae* clone in China, with more numerous genes that allow for tremendous adaptation and transmission throughout the hospital and community (23, 24). Among the 13 strains of Hv-CRKP in this study, there were 12 strains of ST11 with serotype K47/K64. It is necessary to pay attention to KPC-2-producing ST11-K47/K64 Hv-CRKP as they act as a serious clinical threat (25). Furthermore, 13.9% (5/36) of the strains belonged to ST23, which has been identified as the most prevalent MLST type of capsule serotype K1 *K. pneumoniae* isolates (26), and our study revealed that all ST23 strains were K1 capsule serotype. In summary, the dispersed STs in this study confirmed the genetic and characteristic variety of the *K. pneumoniae* isolates, indicating that these strains did not exhibit clonal and cluster transmission features.

Antimicrobial resistance and virulence were thought to be important factors in disease development. In this study, most *K. pneumoniae* isolates were extremely resistant to a wide range of antimicrobial agents, including β-lactamase inhibitors, third generation cephalosporins, and carbapenems, with a resistance rate that was greater than that reported from Taiwan and Beijing (6, 27). Fortunately, all of the 36 *K. pneumoniae* isolates were susceptible to polymyxins and tigecycline. WHO classified carbapenem-resistant *K. pneumoniae* (CRKP) as a critical priority tier of pathogens and the highest priority in novel antimicrobial development in 2017 (8). In China, KPC is the most prevalent genetic mechanism of carbapenem resistance in *K. pneumoniae* species, and it is frequently coupled with other forms of β-lactamases (e.g., ESBLs) (7, 9, 28, 29). In this study, we discovered the co-existence of KPC gene and ESBLs genes. Furthermore, distinct carbapenemase genes in *K. pneumoniae* are frequently coupled with mobile structures, including insertion sequences (IS), plasmids, and transposons, which can accelerate resistance transmission (30), so it should be closely monitored in the medical environment.

Traditionally, the HvKP was characterized by a positive string test or a substantial contributor marker to virulence (31), but this is not totally right. In this study, we identified nine strains as hypermucoviscosity phenotype, and the consistency between hypervirulence and hypermucoviscosity phenotype was only 20.8% (5/24), indicating that the string test performed suboptimally to define HvKP, and will result in substantially more erroneous classifications than with virulence genes detection, which is consistent with other studies (12, 32). Recently, genetic research revealed elevated levels of capsule and siderophore production (predominantly *aerobactin*) as virulence features, and the combination of *peg-344* and *iucA* effectively raised HvKP definition accuracy to 0.98 (sensitivity 0.94 and specificity 1.0) (12, 33). According to one study, the virulence genes, *peg-344, iroB, iucA, rmpA*, and *rmpA*2, all had diagnostic accuracy more than 0.95 for identifying HvKP strains (7, 12). Among these virulence genes, *rmpA* and *rmpA*2 act as mucoid phenotype regulators through enhanced capsule production (33, 34) and were found in 69.4 (25/36) and 55.6% (20/36) of the 36 isolates in this study, respectively. Some *rmpA* and *rmpA*2-positive strains, however, did not exhibit hypermucoviscosity phenotype (data not shown), indicating that alternative regulatory mechanisms for hypermucoviscosity expression may exist. *Peg-344* functions as an inner membrane transporter and is identified on the HvKP virulence plasmid, which can improve the potential of HvKP to spread (35). In our study, 69.4% (25/36) of the isolates tested positive for the *peg-344* gene; salmochelin biosynthetic gene, *iroB*, synthesizing salmonella siderophores, being associated with *K. pneumoniae* virulence (12, 36), was found in 44.4% (16/36) of the 36 isolates in this study. *Aerobactin* is essential for *K. pneumoniae* growth and virulence in the host *via* regulation of iron supply, which could serve as a potential anti-virulence target (34, 37), and our study found that up to 66.7% (24/36) of the isolates were *aerobactin*-positive, as confirmed by *iucA*, one of the genes in *iuc* operon encoding for *aerobactin*.

We then studied the antimicrobial resistance and virulence characteristics that were associated with diverse prognosis. There was no statistically significant difference in patient mortality between CRKP and CSKP groups, and nor between HvKP and cKP groups. However, as compared to the other cases (non-Hv-CRKP group, 56.5 %, 13/23), patients with *K. pneumoniae* meningitis in the Hv-CRKP group had a significantly greater mortality rate (92.3 %, 12/13), indicating a synergistic deadly impact of carbapenem resistance and hypervirulence. Similarly, one study previously demonstrated a 100% fatal outbreak of carbapenem-resistant hypervirulent *K. pneumoniae* isolated from five patients with pneumonia in China (38). To make matters worse, it has been demonstrated that *K. pneumoniae* strains causing meningitis can cause plasmid-mediated resistance and virulence transmission, plasmids encoding resistance to kanamycin, tobramycin, and ampicillin could be conjugated to *Escherichia coli* (39), and that plasmids harboring various virulence genes including *rmpA, rmpA2, aerobactin*, and salmochelin could disseminate to other strains and sublineages (40). Furthermore, plasmids containing *bla*_KPC − 2_ in carbapenem-resistant *K. pneumoniae* (CRKP) of sequence type 11 (ST11) can be horizontally transferred between other species *via* conjugation (41). If this condition continues to deteriorate, Hv-CRKP might become a true global “superbug” posing a major threat to public health (42). As a result, early and precise diagnosis and treatment of Hv-CRKP is extremely beneficial in lowering patient mortality.

Our study elaborated the clinical, microbiological, and molecular epidemiological characteristics of *K. pneumoniae* isolated from 36 meningitis cases, although it has several limitations. First, it was single-center research with a small number of patients included retrospectively enrolled limited cases, which might restrict epidemiological scope and raise selection and information biases, and it even influenced us to investigate the independent risk factors for death from *K. pneumoniae* meningitis. Second, because there are no reference standards for HvKP identification, additional reliable *in vivo* tests (such as mice models and neutrophil phagocytosis assay) may be required to define HvKP strains.

## Conclusion

Our findings showed that antimicrobial resistance and hypervirulence are common in *K. pneumoniae* strains recovered from bacterial meningitis patients, as well as the increased mortality caused by Hv-CRKP strains. Greater innovation and investment in developing several novel techniques to tackle these life-threatening infections will be necessary in future.

## Data availability statement

The original contributions presented in the study are included in the article/Supplementary material, further inquiries can be directed to the corresponding authors.

## Ethics statement

The studies involving human participants were reviewed and approved by the Ethics Committee of the First Affiliated Hospital of Wenzhou Medical University. Written informed consent from the participants' legal guardian/next of kin was not required to participate in this study in accordance with the national legislation and the institutional requirements.

## Author contributions

NH, HJ, and BZ conceived of the presented idea. CZ, JC, and WL collected the data. NH, SL, and JY drafted the manuscript. LW, LiqC, and LijC were responsible for the statistical evaluation. NH, TZ, and JY contributed to the interpretation of the results. All authors have approved the final manuscript.

## Funding

This study was supported by the research grants from the Health Department of Zhejiang Province of the People's Republic of China (no. 2019KY098), the National Natural Science Foundation of China (nos. 81971986 and 82072347), and Key Laboratory of Clinical Laboratory Diagnosis and Translational Research of Zhejiang Province (no. 2022E10022).

## Conflict of interest

The authors declare that the research was conducted in the absence of any commercial or financial relationships that could be construed as a potential conflict of interest.

## Publisher's note

All claims expressed in this article are solely those of the authors and do not necessarily represent those of their affiliated organizations, or those of the publisher, the editors and the reviewers. Any product that may be evaluated in this article, or claim that may be made by its manufacturer, is not guaranteed or endorsed by the publisher.
