# Supplementary material for: Hypervirulent carbapenem-resistant Klebsiella pneumoniae causing highly fatal meningitis in southeastern China
Source: Front Public Health. 2022 Oct 17;10:991306. doi: 10.3389/fpubh.2022.991306 (PMC9621088; doi:10.3389/fpubh.2022.991306)
Supplement: Supplementary file 1 [file Data_Sheet_1.docx]

Supplementary Material

# Supplementary Figure


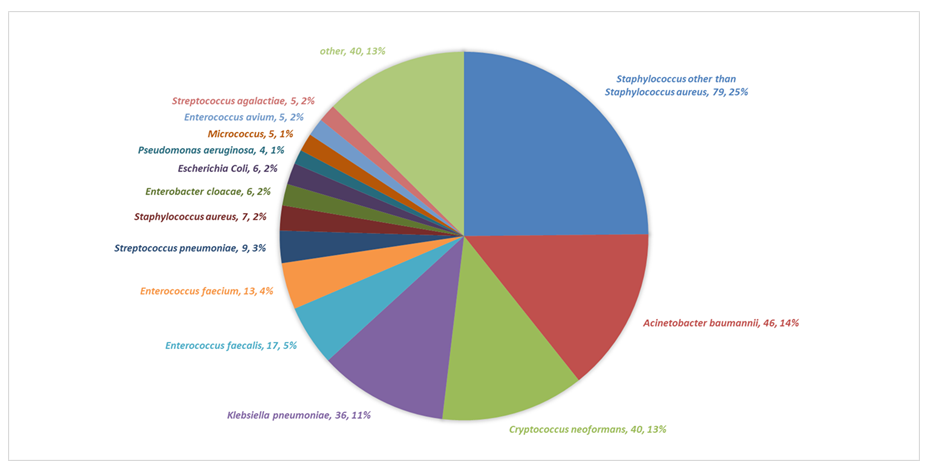


**Supplementary Figure 1 |** The distribution of all meningitis-causing species from 2014 to 2020.


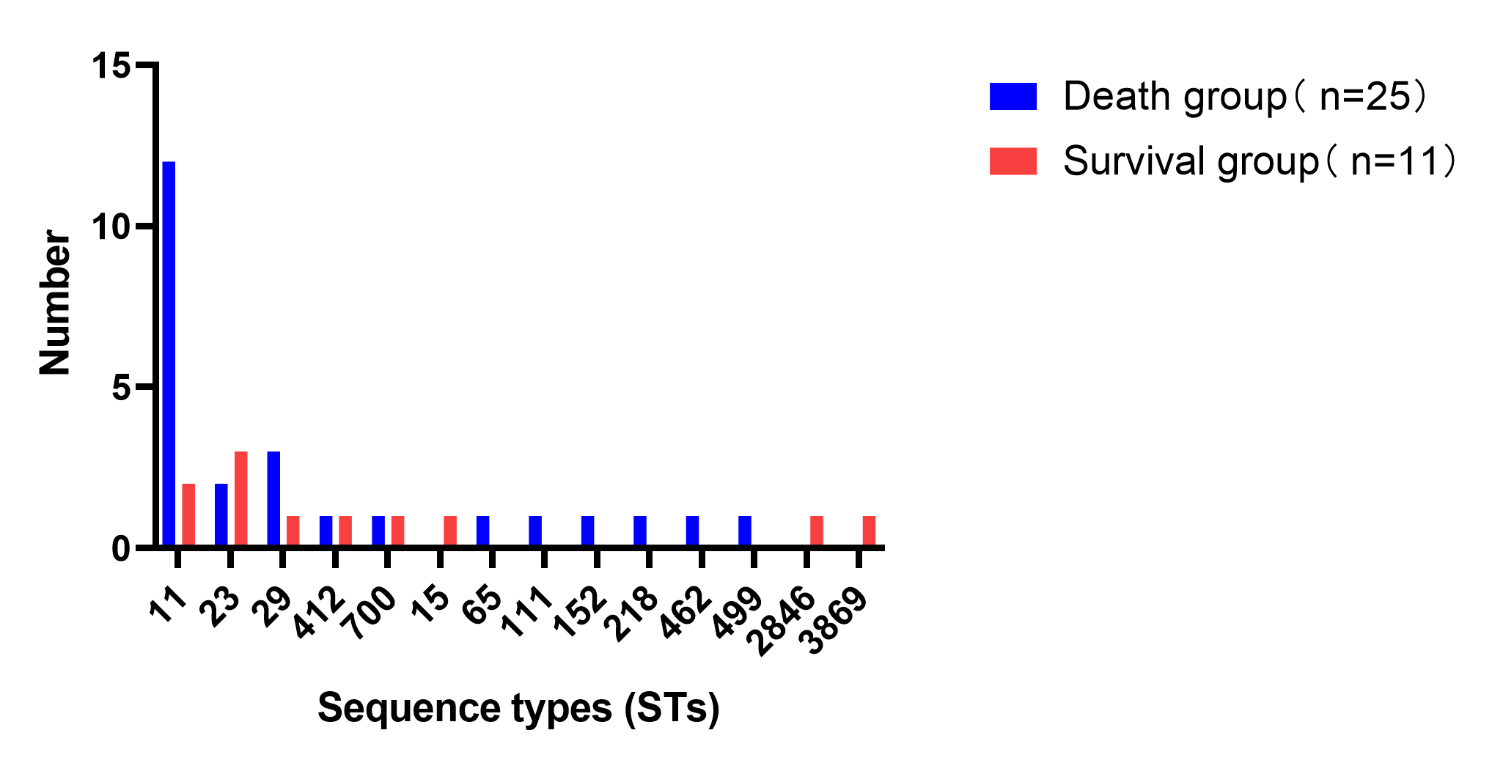


**Supplementary Figure 2 |** Sequence types (STs) distribution of the strains in death group and survival group.

# Supplementary Tables

**Supplementary Table 1 |** Primers, product size, and PCR conditions used for this study.

| **Capsular serotypes** | **Primers** | **bp** | **Temperature (°C)** |  | **Reference** |
| --- | --- | --- | --- | --- | --- |
| wzi | F: CGAGCGCTTTCTATCTTGGT | 580 | 55 |  | [1] |
|  | R: GAGAGCCACTGGTTCCAGAA |  |  |  |  |
| Virulence Gene | | | |  |  |
| *iucA* | F1: AATCAATGGCTATTCCCGCTG | 239 | 59 |  | [2] |
|  | R1: CGCTTCACTTCTTTCACTGACAGG |  |  |  |  |
|  | F2: GCTTATTTCTCCCCAACCC | 583 | 59 |  |  |
|  | R2: TCAGCCCTTTAGCGACAAG |  |  |  |  |
| *iroB* | F1: ATCTCATCATCTACCCTCCGCTC | 235 | 59 |  | [2] |
|  | R1: GGTTCGCCGTCGTTTTCAA |  |  |  |  |
|  | F2: CAAAAAAGCAGCAGAGGC | 585 | 59 |  |  |
|  | R2: TCACTGGCGGAATCCAACAC |  |  |  |  |
| *peg-344* | F1: CTTGAAACTATCCCTCCAGTC | 508 | 53 |  | [2] |
|  | R1: CCAGCGAAAGAATAACCCC |  |  |  |  |
|  | F2: AAAGGACAGAAAGCCAGTG | 411 | 53 |  |  |
|  | R2: CAATGACGAGGGGGATAATC |  |  |  |  |
| *_p_rmpA* | F: GAGTAGTTAATAAATCAATAGCAAT | 332 | 50 |  | [2] |
|  | R: CAGTAGGCATTGCAGCA |  |  |  |  |
| *_p_rmpA2* | F: GTGCAATAAGGATGTTACATTA | 430 | 50 |  | [2] |
|  | R: GGATGCCCTCCTCCTG |  |  |  |  |
| *_c_rmpA* | F: GTAATAGAGATATAAATATCATATTGA | 588 | 50 |  | [2] |
|  | R: CATCTTTCATCAACCATTTC |  |  |  |  |
| ESBL Gene | | | |  |  |
| *bla*_CTX-M-1_ | F: GGTTAAAAAATCACTGCGTC | 825-863 | 48 |  | [3] |
|  | R: TTGGTGACGATTTTAGCCGC |  |  |  |  |
| *bla*_CTX-M-2_ | F: ATGATGACTCAGAGCATTCG | 827-865 | 50 |  | [3] |
|  | R: TGGGTTACGATTTTCGCCGC |  |  |  |  |
| *bla*_CTX-M-8_ | F: ATGGTACGACGAATGATATC | 246 | 48 |  | [4] |
|  | R: TAATCATACAGAAGTCGCAG |  |  |  |  |
| *bla*_CTX-M-9_ | F: ATGGTGACAAAGAGAGTGCA | 863-826 | 50 |  | [3] |
|  | R: CCCTTCGGCGATGATTCTC |  |  |  |  |
| *bla*_CTX-M-25_ | F: ATGATGAGAAAAAGCGTAAG | 869 | 50 |  | [5] |
|  | R: TTAATAACCGTCGGTGAC |  |  |  |  |
| *bla*_CTX-M-65_ | F: GCAGTACAGCGACAATACCG | 320 | 50 |  | [6] |
|  | R: TATCACCCACAGTCCACGAC |  |  |  |  |
| *bla*_SHV_ | F: AACGCTTTCCCATGATGAGC | 322 | 55 |  | [6] |
|  | R: CGCCTCATTCAGTTCCGTTT |  |  |  |  |
| *bla*_TEM_ | F: GGGAACCGGAGCTGAATGAA | 254 | 59 |  | [6] |
|  | R: CAGTGCTGCAATGATACCGC |  |  |  |  |
| Carbapenem resistance gene | | | |  |  |
| *bla*_KPC-2_ | F: CGCCAATTTGTTGCTGAAGG | 341 | 55 |  | [6] |
|  | R: CATAGTCATTTGCCGTGCCA |  |  |  |  |
| *bla*_NDM-1_ | F: TCTCGACATGCCGGGTTTCGG | 475 | 57 |  | [7] |
|  | R: ACCGAGATTGCCGAGCGACTT |  |  |  |  |
| *bla*_OXA-48_ | F: CGCATCTTGTTGTCCAAGTG | 1012 | 52 |  | [8] |
|  | R: TCGAGCATCAGCATTTTGTC |  |  |  |  |
| *bla*_SPM_ | F: CCTACAATCTAACGGCGACC | 649 | 55 |  | [9] |
|  | R: TCGCCGTGTCCAGGTATAAC |  |  |  |  |
| *bla*_VIM_ | F: GGTCTCATTGTCCGTGATGGTGATGAG  R: CTCGATGAGAGTCCTTCTAGAG | 271 | 50 |  | [10] |
| Membrane porins encode resistance genes |  |  |  |  |  |
| *OmpK35* | F: CAGGTCCTTGCCTTTGGTCT | 283 | 60 |  | [11] |
|  | R: CAACGGTATCGCACTGTCTG |  |  |  |  |
|  |  |  |  |  |  |
|  |  |  |  |  |  |
|  |  |  |  |  |  |
| *OmpK36* | F: GCAAAGCCCAGGGAACC | 254 | 60 |  | [11] |
|  | R: CGTACCGCCTTGAAACAGA |  |  |  |  |
| *OmpK37* | F: GGCGATTACGGCTCCTT | 265 | 60 |  | [11] |
|  | R: TGCTGCGGTTATTGGTG |  |  |  |  |

References:

[1] Fan Q, Shen L, Hu R. Application of wzi sequencing in the detection of serotypes of Klebsiella pneumoniae carried in feces. Journal of Clinical and Pathology. 2020, 40(11): 2844-2850. doi: 10.3978/j.issn.2095-6959.2020.11.007.

[2] Russo TA, MacDonald U. The Galleria mellonella Infection Model Does Not Accurately Differentiate between Hypervirulent and Classical Klebsiella pneumoniae. mSphere. 2020;5(1):e00850-19. Published 2020 Jan 8. doi:10.1128/mSphere.00850-19

[3] M. Saladin, et al.: Diversity of CTX-M beta-lactamases and their promoter regions from Enterobacteriaceae isolated in three Parisian hospitals. FEMS Microbiol Lett. 209(2), 161-8 (2002). doi:10.1111/j.1574-6968.2002.tb11126.x

[4] C. J. Munday, J. Xiong, C. Li, D. Shen and P. M. Hawkey: Dissemination of CTX-M type beta-lactamases in Enterobacteriaceae isolates in the People's Republic of China. Int J Antimicrob Agents. 23(2), 175-80 (2004). doi:10.1016/j.ijantimicag.2003.07.004

[5] M. Cartelle, et al.: Characterisation of the first CTX-M-10-producing isolate of Salmonella enterica serotype Virchow. Clin Microbiol Infect. 12(3), 285-7 (2006). doi:10.1111/j.1469-0691.2005.01300.x

[6] Zhao Y, Zhang X, Torres VVL, et al. An Outbreak of Carbapenem-Resistant and Hypervirulent Klebsiella pneumoniae in an Intensive Care Unit of a Major Teaching Hospital in Wenzhou, China. Front Public Health. 2019;7:229. doi:10.3389/fpubh.2019.00229

[7] L. Hidalgo, et al.: Association of the novel aminoglycoside resistance determinant RmtF with NDM carbapenemase in Enterobacteriaceae isolated in India and the UK. J Antimicrob Chemother. 68(7), 1543-50 (2013). doi:10.1093/jac/dkt078

[8] L. Ma, et al.: Emergence of OXA-48-Producing Klebsiella pneumoniae in Taiwan. PLoS One. 10(9), e0139152 (2015). doi:10.1371/journal.pone.0139152

[9] G. Yu, et al.: First report of novel genetic array aacA4-blaIMP-25-oxa30-catB3 and identification of novel metallo-beta-lactamase gene blaIMP25: A Retrospective Study of antibiotic resistance surveillance on Psuedomonas aeruginosa in Guangzhou of South China, 2003-2007. Microb Pathog. 95, 62-67 (2016). doi:10.1016/j.micpath.2016.02.021

[10] F. M. Kaczmarek, F. Dib-Hajj, W. Shang and T. D. Gootz: High-level carbapenem resistance in a Klebsiella pneumoniae clinical isolate is due to the combination of bla(ACT-1) beta-lactamase production, porin OmpK35/36 insertional inactivation, and down-regulation of the phosphate transport porin phoe. Antimicrob Agents Chemother. 50(10), 3396-406 (2006). doi:10.1128/AAC.00285-06

[11] Pan X, Liu H, Ji Q. Penicillium bloodstream infections resistant to carbon alkene Klebsiella pneumoniae bacteria strain characteristics analysis. Journal of Chinese medicine and clinical. 2021, 21 (7): 1082-1084. doi: 10.11655 / zgywylc2021.07.007.

**Supplementary Table 2 |** Characteristics of 36 *Klebsiella pneumoniae* meningitis patient.

| **Patient**  **no.** | **Infection type** | **Strain**  **ID** | **Age** | **Sex** | **Unit** | **Length of**  **stay (Days)** | | **Main diagnosis** | **Extrameningeal infections** | **MDR strains** | **Antimicrobial**  **use** | **Outcome** |
| --- | --- | --- | --- | --- | --- | --- | --- | --- | --- | --- | --- | --- |
| P1 | HA | **FK1819** | 29 | Male | Neurosurgery | | 52 | Subdural hematoma | Pneumonia | Yes | MEM, CSL, VA, CAZ, CHL | Died |
| P2 | HA | **FK2262** | 16 | Male | Trauma surgery | | 22 | Multiple injuries | Pneumonia, BSI | Yes | MEM, VA, CSL, TGC | Died |
| P3 | HA | **FK2357** | 16 | Male | ICU | | 17 | Cerebral hemorrhage | Pneumonia, BSI | Yes | CSL, CXM, CAZ, FEP, LNZ | Died |
| P4 | HA | **FK2682** | 47 | Male | Neurosurgery | | 51 | Brain contusion and herniation | Pneumonia, BSI | Yes | PIP, MEM, VA | Died |
| P5 | HA | **FK2784** | 38 | Male | ICU | | 4 | Coma (to be investigated) | Pneumonia | Yes | VA, PIP, CSL, FOS, IPM, SXT | Died |
| P6 | HA | **FK2877** | 52 | Male | Neurosurgery | | 20 | Brain herniation | BSI | Yes | CXM, PIP, VA | Died |
| P7 | HA | **FK3092** | 67 | Female | Neurosurgery | | 14 | Intracranial aneurysm | Pneumonia, BSI | Yes | IPM, FOS, SXT, VA, MEM | Died |
| P8 | CA | **FK3616** | 59 | Male | ICU | | 2 | Coma, Intracranial infection | / | Yes | IPM, LNZ | Died |
| P9 | CA | **FK3642** | 56 | Male | ICU | | 5 | Head trauma | Pneumonia, BSI | Yes | IPM, LNZ, FOS | Died |
| P10 | HA | **FK4724** | 51 | Male | ICU | | 16 | Multiple injuries | Pneumonia, BSI | Yes | PIP, MEM, LNZ | Died |
| P11 | HA | **FK5164** | 60 | Male | Neurosurgery | | 53 | Thalamic hematoma | Pneumonia, BSI | Yes | PIP, CSL, MEM, IPM, VA, LNZ, PB | Died |
| P12 | HA | **FK5222** | 34 | Male | Neurosurgery | | 58 | Brain contusion | Pneumonia, BSI | Yes | PIP, VA, LNZ, MEM, TGC | Survival |
| P13 | HA | **FK7117** | 52 | Male | ICU | | 13 | Multiple injuries | Pneumonia, BSI | Yes | AMC | Died |
| P14 | HA | FK1541 | 57 | Male | ICU | | 25 | Suppurative meningitis | Pneumonia | No | IPM, VA, FOS | Died |
| P15 | HA | FK1689 | 41 | Male | Neurosurgery | | 17 | Intracranial infection | / | Yes | PIP, IPM, VA | Died |
| P16 | CA | FK1916 | 62 | Male | ICU | | 2 | Intracranial infection | Pneumonia | No | IPM | Died |
| P17 | HA | FK1971 | 59 | Male | ICU | | 4 | Suppurative meningitis | / | No | IPM | Died |
| P18 | CA | FK2652 | 63 | Male | ICU | | 37 | Suppurative meningiti | Pneumonia | No | MEM, IPM, VA, LNZ, FOS | Survival |
| P19 | CA | FK2686 | 69 | Male | ICU | | 30 | Intracranial infection | Pneumonia | Yes | VA, PIP, IPM, SXT | Died |
| P20 | HA | FK3150 | 48 | Female | Neurosurgery | | 23 | Cranial defect repair | / | No | IPM, VA | Survival |
| P21 | CA | FK3680 | 63 | Male | ICU | | 17 | Intracranial infection | Pneumonia | No | IPM, LNZ, FOS | Died |
| P22 | CA | FK3907 | 54 | Male | ICU | | 19 | sepsis | / | No | MEM | Survival |
| P23 | HA | FK4006 | 57 | Male | ICU | | 56 | Head trauma | / | No | CAZ, MEM, CSL, LNZ | Died |
| P24 | HA | FK4956 | 53 | Male | ICU | | 20 | Suppurative meningitis | Pneumonia | No | MEM, LNZ | Survival |
| P25 | HA | FK5223 | 56 | Female | Neurosurgery | | 59 | Intracranial infection | Pneumonia | Yes | CAZ, MEM, LNZ, FOS | Survival |
| P26 | HA | FK6048 | 50 | Male | ICU | | 9 | Suppurative meningitis | / | No | CAZ, MEM, LNZ, FOS | Died |
| P27 | HA | FK6696 | 52 | Male | Infectious Department | | 8 | Intracranial infection | Pneumonia | Yes | CAZ, MEM, LNZ | Survival |
| P28 | HA | FK7178 | 66 | Female | Neurosurgery | | 48 | Intracranial infection | / | No | CAZ, MEM, LNZ | Survival |
| P29 | CA | FK7427 | 52 | Female | ICU | | 3 | Intracranial infection | BSI | No | MEM | Died |
| P30 | HA | FK7521 | 62 | Male | Department of Stomatology | | 34 | Intracranial infection | / | No | CAZ, MEM, LNZ | Survival |
| P31 | CA | FK7664 | 42 | Female | Neurosurgery | | 45 | Brain abscesses | / | No | MEM, LNZ | Survival |
| P32 | HA | FK8534 | 36 | Male | ICU | | 23 | Intracranial infection | BSI | No | MEM, LNZ | Died |
| P33 | HA | FK8682 | 70 | Male | ICU | | 8 | septic shock | BSI | No | MEM, LNZ | Died |
| P34 | HA | FK8716 | 79 | Female | ICU | | 1 | Intracranial infection | BSI | No | / | Died |
| P35 | CA | FK8848 | 76 | Male | Neurosurgery | | 36 | Brain abscesses | / | No | CAZ, MEM, LNZ, | Survival |
| P36 | HA | FK9304 | 25 | Male | ICU | | 39 | Suppurative meningitis | / | No | CAZ, MEM, LNZ, FOS | Died |

Abbreviations: Bold strain number are indicating hypervirulent-carbapenem resistance *K. pneumoniae* (Hv-CRKP) strain. HA, hospital acquired infection; CA, community acquired infection; BSI, bloodstream infection; MDR, multidrug resistant; ICU, intensive care unit; IPM, imipenem; MEM, meropenem; CSL, cefoperazone-sulbactam; VA, vancomycin; CAZ, ceftazidime; CHL, chloramphenicol; TGC, tigecycline; CXM, cefuroxime; FEP, cefepime; LNZ, linezolid; PIP, piperacillin; FOS, Fosfomycin; SXT, trimethoprim-sulfamethoxazole; AMC, amoxicillin and clavulanic acid; PB, polymyxin B; AMK, amikacin.

**Supplementary Table 3 |** Comparative analysis between the Hv-CRKP group and the non- Hv-CRKP group.

| **Clinical characteristics** | **Total (*n* = 36)** | **Available patients or isolates (*n* = 36)** | | | ***P*-values** |
| --- | --- | --- | --- | --- | --- |
|  |  | **Hv-CRKP group**  **(*n* = 13)** | **Non- Hv-CRKP group**  **(*n* = 23)** | |  |
| Demographic data | | | | | |
| Age (years), median (IQR) | 54, (44, 62) | 51, (34, 56) | | 57, (51, 63) | **0.034** |
| Gender, n (%) |  |  | |  |  |
| Male | 29 (80.6) | 12 (92.3) | | 17 (73.9) | 0.187 |
| Female | 7 (19.4) | 1 (7.7) | | 6 (26.1) |  |
| Underlying conditions, n (%) | | | | | |
| Hypertension | 13 (36.1) | 3 (23.1) | | 10 (43.6) | 0.292 |
| Diabetes mellitus | 12 (33.3) | 1 (7.7) | | 11 (47.8) | **0.025** |
| History of surgery | 11 (30.6) | 3 (23.1) | | 8 (34.8) | 0.708 |
| Head trauma | 11 (30.6) | 7 (53.8) | | 4 (17.4) | 0.060 |
| Clinical Symptoms, n (%) | | | | | |
| Fever | 11 (30.6) | 3 (23.1) | | 8 (34.8) | 0.708 |
| Headache | 8 (22.2) | 3 (23.1) | | 5 (21.7) | 1.000 |
| Vomiting | 11 (30.6) | 4 (30.8) | | 7 (30.4) | 1.000 |
| Unconscious | 19 (52.8) | 8 (61.5) | | 11 (47.8) | 0.502 |
| Muscle tone changes | 12 (33.3) | 7 (53.8) | | 5 (21.7) | 0.071 |
| Length of stay (Days), median (IQR) | 20 (8, 38) | 17 (13, 51) | | 23 (8, 37) | 0.081 |
| Extrameningeal infections, n (%) | | | | | |
| Bacteremia | 14 (38.9) | 7 (53.8) | | 7 (30.4) | 0.286 |
| Pneumonia | 19 (52.8) | 9 (69.2) | | 10 (43.5) | 0.177 |
| Liver abscess | 2 (5.6) | 0 | | 2 (8.7) | 0.525 |
| Brain abscess | 6 (16.7) | 2 (15.4) | | 4 (17.4) | 1.000 |
| Stay in ICU | 21 (58.3) | 7 (53.8) | | 14 (60.9) | 0.736 |
| Mechanical ventilation | 27 (75.0) | 10 (76.9) | | 17 (74.0) | 1.000 |
| GCS, median (IQR) | 12.5（1, 14.50） | 14（11, 15） | | 7（6, 13） | 0.653 |
| Inflammation index, median (IQR) | | | | | |
| White blood cell count (WBC,10^9 /L) | 12.44 (9.72, 14.82) | 13.05 (10.39, 13.85) | | 12.17 (8.59, 17.75) | 0.922 |
| Procalcitonin (PCT, ug/L) | 1.15 (0.14, 12.18) | 0.54 (0.15, 1.875) | | 6.68 (0.17, 18.57) | 0.344 |
| C reactive protein (CRP, mg/L) | 90.00 (72.40, 90) | 90 (55.55, 90) | | 76.25 (28, 90) | 0.289 |
| Percentage of neutrophils | 0.87 (0.83, 0.91) | 0.86 (0.84, 0.91) | | 0.87 (0.79, 0.90) | 0.724 |
| CSF profiles, median (IQR) | | | | | |
| RBC count (per µL) | 590 (111, 2017) | 920 (163, 1540) | | 400 (120, 2070) | 0.755 |
| WBC count (per µL) | 3500 (126, 19600) | 4000 (66, 13680) | | 3000 (300, 26400) | 0.725 |
| Sugar (mmol/L) | 1.11 (1.11, 3.40) | 1.65 (1.11, 3.7) | | 1.11 (1.11, 2.9) | 0.373 |
| Chlorine (mmol/L) | 116 (110, 121) | 117 (111, 120) | | 116 (110, 123) | 0.650 |
| Protein (g/L) | 6000  (2934, 13961) | 6161  (3626, 12029) | | 6000  (2868, 14259) | 0.693 |

Abbreviations: ICU, Intensive Care Unit; GCS, Glasgow Coma Scale; RBC, Red Blood Cell; WBC, White Blood Cell.

**Supplementary Table 4 |** Minimum inhibitory concentrations (MICs) of 36 *Klebsiella pneumoniae* isolates.

| **Isolates** | **Year** | **MIC (μg/mL)** | | | | | | | | | | | | | | | |
| --- | --- | --- | --- | --- | --- | --- | --- | --- | --- | --- | --- | --- | --- | --- | --- | --- | --- |
|  |  | **AMP** | **CRO** | **CAZ** | **FEP** | **NIT** | **CIP** | **LEV** | **GEN** | **TOB** | **SXT** | **FOS** | **ETP** | **IPM** | **MEM** | **COL** | **TGC** |
| **FK1541** | 2014 | >128 | 0.0625 | 0.25 | 1 | 64 | 0.0625 | 0.125 | 1 | 1 | 2/38 | 64 | ＜0. 5 | 0.25 | 0.0078 | 0.0625 | 0.5 |
| **FK1689** | 2014 | >128 | >64 | 2 | >64 | >512 | >8 | 8 | >64 | 16 | >64/1216 | >1024 | ＜0.5 | 0.5 | 0.25 | 0.03125 | 0.5 |
| **FK1819^a, b^** | 2014 | >128 | >64 | >64 | >64 | 256 | >8 | >16 | >64 | >64 | 2/38 | >1024 | >16 | 32 | >32 | 0.03125 | 0.5 |
| **FK1916^a^** | 2015 | >128 | >64 | >64 | 1 | 64 | 0.0625 | 0.5 | 1 | 1 | 4/76 | 64 | ＜0.5 | 1 | 1 | 0.0625 | 1 |
| **FK1971^a^** | 2015 | >128 | 2 | >64 | 4 | 256 | 0.03125 | 0.125 | 4 | 4 | 8/152 | 1024 | ＜0.5 | 0.25 | 0.0078 | 0.0625 | 0.5 |
| **FK2262^a, b^** | 2015 | >128 | >64 | >64 | >64 | 256 | >8 | >16 | >64 | >64 | 2/38 | >1024 | >16 | 32 | >32 | 0.03125 | 0.25 |
| **FK2357^a, b^** | 2015 | >128 | >64 | >64 | >64 | 256 | >8 | >16 | >64 | >64 | 2/38 | >1024 | >16 | 32 | >32 | 0.03125 | 0.5 |
| **FK2682^a, b^** | 2015 | >128 | >64 | >64 | >64 | 256 | >8 | >16 | 64 | 16 | >64/1216 | >1024 | >16 | >64 | >32 | 0.03125 | 2 |
| **FK2686^b^** | 2015 | >128 | >64 | >64 | >64 | 512 | >8 | >16 | >64 | >64 | >64/1216 | >1024 | >16 | 64 | >32 | 0.0625 | 1 |
| **FK2784^a, b^** | 2016 | >128 | >64 | >64 | >64 | >512 | >8 | >16 | >64 | >64 | >64/1216 | >1024 | >16 | 32 | >32 | 0.0625 | 2 |
| **FK2877^a, b^** | 2016 | >128 | >64 | >64 | >64 | >512 | >8 | >16 | >64 | >64 | >64/1216 | >1024 | >16 | 64 | >32 | 0.0625 | 2 |
| **FK3092^a, b^** | 2016 | >128 | >64 | >64 | >64 | 512 | >8 | >16 | >64 | >64 | 16/304 | >1024 | >16 | >64 | >32 | 0.03125 | 2 |
| **FK3616^a, b^** | 2016 | 32 | 0.25 | 0.5 | 1 | 256 | 0.5 | 2 | 1 | 1 | 16/304 | 64 | >16 | 0.25 | 0.25 | 0.0625 | 2 |
| **FK3642^a, b^** | 2017 | >128 | >64 | >64 | >64 | 256 | >8 | >16 | >64 | >64 | 4/76 | 64 | >16 | >64 | >32 | 0.03125 | 2 |
| **FK3680^a^** | 2017 | 32 | 2 | 1 | 4 | 256 | 0.03125 | 0.125 | 4 | 1 | 2/38 | 1024 | ＜0.5 | 1 | 0.078 | 0.0625 | 1 |
| **FK4006** | 2017 | >128 | >64 | 2 | 8 | 64 | 1 | 2 | >64 | 8 | >64/1216 | 64 | ＜0.5 | 0.25 | 0.078 | 0.0625 | 1 |
| **FK4724^a, b^** | 2017 | >128 | >64 | >64 | >64 | 256 | >8 | >16 | >64 | >64 | 16/304 | >1024 | >16 | >64 | >32 | 0.03125 | 0.5 |
| **FK5164^a, b^** | 2018 | >128 | >64 | >64 | >64 | 256 | >8 | >16 | >64 | >64 | >64/1216 | >1024 | >16 | 64 | >32 | 0.03125 | 1 |
| **FK6048^a^** | 2018 | 32 | >64 | 1 | 0.5 | 128 | 0.5 | 2 | 0.5 | 0.5 | 4/76 | 1024 | ＜0.5 | 0.25 | 0.0078 | 0.0625 | 1 |
| **FK7117^a, b^** | 2019 | >128 | >64 | >64 | >64 | 512 | >8 | >16 | >64 | >64 | >64/1216 | >1024 | >16 | >64 | >32 | 0.03125 | 2 |
| **FK7427** | 2019 | 16 | 1 | 1 | 1 | 32 | 0.25 | 0.25 | 1 | 1 | 1/19 | 32 | ＜0.5 | 0.5 | 0.25 | 0.0625 | 0.5 |
| **FK8534** | 2020 | 16 | 1 | 1 | 1 | 32 | 0.25 | 0.25 | 1 | 1 | 1/19 | 32 | ＜0.5 | 0.5 | 0.25 | 0.0625 | 0.5 |
| **FK8682^a^** | 2020 | 32 | 1 | 0.5 | 1 | 32 | 0.25 | 0.125 | 0.5 | 0.5 | 2/38 | 32 | ＜0.5 | 1 | 0.25 | 0.0625 | 0.5 |
| **FK8716** | 2020 | 16 | 1 | 1 | 1 | 32 | 0.25 | 0.25 | 1 | 1 | 1/19 | 32 | ＜0.5 | 0.5 | 0.25 | 0.0625 | 0.5 |
| **FK9304^a^** | 2020 | 16 | 1 | 1 | 1 | 32 | 0.25 | 0.25 | 1 | 1 | 1/19 | 32 | ＜0.5 | 0.5 | 0.25 | 0.03125 | 0.5 |
| FK2652^a^ | 2015 | >128 | >64 | >64 | >64 | 256 | 0.03125 | 0.125 | >64 | >64 | 4/76 | 1024 | ＜0.5 | 0.25 | 0.0078 | 0.0625 | 0.5 |
| FK3150 | 2016 | >128 | 8 | >64 | >64 | 128 | 0.03125 | 0.125 | >64 | >64 | 4/76 | >1024 | ＜0.5 | 1 | 0.25 | 0.0625 | 0.5 |
| FK3907 | 2017 | >128 | >64 | >64 | >64 | 512 | 0.5 | 1 | >64 | >64 | >64/1216 | 1024 | ＜0.5 | 0.25 | 0.0078 | 0.0625 | 1 |
| FK4956 | 2018 | >128 | 1 | 2 | >64 | 64 | 0.03125 | 0.125 | 16 | 4 | 2/38 | >1024 | ＜0.5 | 1 | 0.25 | 0.0625 | 0.5 |
| FK5222^a, b^ | 2018 | >128 | >64 | >64 | >64 | 512 | >8 | >16 | >64 | >64 | >64/1216 | >1024 | >16 | >64 | >32 | 0.0625 | 2 |
| FK5223^b^ | 2018 | >128 | >64 | >64 | >64 | 512 | >8 | >16 | >64 | >64 | >64/1216 | >1024 | >16 | 16 | >32 | 0.0625 | 2 |
| FK6696^b^ | 2019 | >128 | >64 | >64 | >64 | 256 | >8 | >16 | >64 | >64 | 1/19 | >1024 | >16 | >64 | >32 | 1 | 1 |
| FK7178^a^ | 2019 | >128 | >64 | 64 | >64 | 512 | >8 | >16 | >64 | >64 | 2/38 | >1024 | ＜0.5 | 1 | 1 | 0.0625 | 0.5 |
| FK7521^a^ | 2019 | 32 | 1 | 0.5 | 1 | 32 | 0.25 | 0.125 | 0.5 | 0.5 | 2/38 | 32 | ＜0.5 | 1 | 0.25 | 0.0625 | 0.5 |
| FK7664^a^ | 2019 | 32 | 0.25 | 0.5 | 0.25 | 32 | 0.25 | 0.125 | 0.5 | 0.5 | 1/19 | 32 | ＜0.5 | 0.25 | 0.0078 | 0.0625 | 0.5 |
| FK8848^a^ | 2020 | 32 | 0.25 | 0.5 | 0.25 | 32 | 0.25 | 0.125 | 0.5 | 0.5 | 1/19 | 32 | ＜0.5 | 0.25 | 0.0078 | 0.03125 | 0.5 |
| ATCC25922 |  | >128 | 2 | 1 | 2 | 64 | 0.015625 | 0.0625 | 2 | 2 | 0.5/9.5 | 64 | ＜0.5 | 0.25 | 0.0078 | 0.03125 | 0.125 |

Abbreviations: Bold strain number are indicating the infected patient is non-survival. AMP, ampicillin; CRO, ceftriaxone; CAZ, ceftazidime; FEP, Cefepime; NIT, nitrofurantoin; CIP, ciprofloxacin; LEV, levofloxacin; GEN, gentamicin; TOB, tobramycin; SXT, sulfamethoxazole; FOS, Fosfomycin; ETP, ertapenem; IPM, imipenem; MEM, meropenem; COL, colistin; TGC, tigecycline. ^a^indicating hypervirulent *K. pneumoniae* (hvKP) strain. ^b^indicating carbapenem resistance strain.
